# Stat3 Signaling Pathway: A Future Therapeutic Target for Bone-Related Diseases

**DOI:** 10.3389/fphar.2022.897539

**Published:** 2022-04-25

**Authors:** Jiadong Li, Zhifeng Yin, Biaotong Huang, Ke Xu, Jiacan Su

**Affiliations:** ^1^ Institute of Translational Medicine, Shanghai University, Shanghai, China; ^2^ School of Medicine, Shanghai University, Shanghai, China; ^3^ School of Life Sciences, Shanghai University, Shanghai, China; ^4^ Department of Orthopedics, Shanghai Zhongye Hospital, Shanghai, China

**Keywords:** stat3, bone-related diseases, target therapeutic, signaling pathway, biological functions

## Abstract

Signal transducer and activator of transcription 3 (Stat3) is activated by phosphorylation and translocated to the nucleus to participate in the transcriptional regulation of DNA. Increasing evidences point that aberrant activation or deletion of the Stat3 plays a critical role in a broad range of pathological processes including immune escape, tumorigenesis, and inflammation. In the bone microenvironment, Stat3 acts as a common downstream response protein for multiple cytokines and is engaged in the modulation of cellular proliferation and intercellular interactions. Stat3 has direct impacts on disease progression by regulating mesenchymal stem cells differentiation, osteoclast activation, macrophage polarization, angiogenesis, and cartilage degradation. Here, we describe the theoretical basis and key roles of Stat3 in different bone-related diseases in combination with *in vitro* experiments and animal models. Then, we summarize and categorize the drugs that target Stat3, providing potential therapeutic strategies for their use in bone-related diseases. In conclusion, Stat3 could be a future target for bone-related diseases.

## Introduction

Bone-related diseases are a group of chronic diseases that are most prevalent in the elderly and obese population, including osteoarthritis (OA), osteoporosis (OP), bone dysplasia and bone defects. As the global population ages, bone-related diseases are often characterized by multiple co-morbidities, are a predominant contributor to the disability of the elderly, and constitute a primary factor in the global health care burden and rising costs to society (Jin et al., 2020; Kirk et al., 2020). Bone formation and restoration in physiological or pathological conditions is mediated by a diversity of intraosseous cells, including osteoblasts secreting bone mineral matrix, osteoclasts resorbing bone, and chondrocytes constituting the cartilage matrix (Salhotra et al., 2020). In various pathological microenvironments, osteoblasts, osteoclasts, chondrocytes, or other cells regulate cellular biological activities by secreting diverse cytokines that influence the disease progression (Hu et al., 2020). The pathogenesis of bone-related diseases is complicated and involves intercellular communication between different cells in the bone microenvironment, which is jointly regulated by multiple cytokines (Safari et al., 2021).

Signal transducer and activator of transcription 3 (Stat3) is a cytoplasmic transcription factor which activates by inflammatory cytokines or growth factors and then translocates to the nucleus, where it is involved in the regulation of DNA transcription (Yu et al., 2009). Stat3 has become a research hotspot because of its essential function during diverse biological processes such as proliferation, differentiation, anti-apoptosis, inflammatory response, and angiogenesis (Cheng et al., 2020; Tian et al., 2020; He et al., 2021). In previous researches, Stat3 was considered as a critical signaling molecule for immune escape, atherosclerosis, malignancy and cardiac injury (Huynh et al., 2017; Chen et al., 2019; Comita et al., 2021; Zhang et al., 2022). Over-activation of Stat3 will lead to poor prognosis and drug resistance in osteosarcoma (Liu et al., 2021). As yet, several studies have shown that Stat3, a downstream pathway co-activated by multiple cytokines, is likely to take an essential role in OA, OP, bone development and repair (Latourte et al., 2017; Yang et al., 2019; Zhou et al., 2021). This indicates that Stat3 signaling pathway may be a prospective target for the therapy of bone-related diseases.

Here, we review the structure and biological function of the Stat3, the regulatory role and therapeutic prospects of targeting Stat3 signaling pathway in bone-related diseases. The purpose of this study is to provide a further reference for novel insights into the treatment of bone-related diseases and the clinical translational application of Stat3 inhibitors.

## Signal Transducer and Activator of Transcription 3 Signaling Pathway

### Structure of Signal Transducer and Activator of Transcription 3

Stat3 is conservedly expressed in eukaryotes, localized on human chromosome 17, and consists of six conserved structural domains (N-terminal domain, coiled-coil domain, DNA binding domain, linker domain, Src homology domain, transactivation domain) (Leonard and O'Shea, 1998). Phosphorylation sites at Tyr705 and Ser727 of the Src homology domain and transactivation domain are intimately interrelated with the activation of Stat3 (Maritano et al., 2004). When cytokines bind to the tyrosine kinase-associated receptor on the cell membrane, they form ligand-receptor complexes and immediately trigger the phosphorylation of the intracellular domain of the coupled Janus kinase (JAK) (Bharadwaj et al., 2020). The phosphorylated tyrosine site on JAK acts as a docking site to recruit Stat3 with the SH2 structural domain for phosphorylation modification, and the phosphorylated Stat3 is translocated to nucleus to bind the genes and regulate transcription (Johnson et al., 2018). In addition, Stat3 has four different isoforms, Stat*α*, Stat*β*, Stat*γ*, and Stat*δ* (Hevehan et al., 2002). During human skeletal development, growth hormone inhibits Runx Family Transcription Factor 2 (Runx2) transcriptional activity by promoting the physical interaction of Stat3*β* with Runx2 in osteoblasts, suggesting that different isoforms of Stat3 may be a pivotal trigger for its functional variability (Ziros et al., 2004).

### Biological Function of Signal Transducer and Activator of Transcription 3 Signaling Pathway

Stat3 was first identified in 1996 by researchers at Rockefeller University in the intracellular transduction of epidermal growth factor (EGF) and interleukin 6 (IL-6), and is commonly believed to be an acute response factor mediating growth factors and inflammatory factors (Zhong et al., 1994). Stat3, as a downstream intracellular effector of inflammatory factors and growth factors, controls cell proliferation, migration, apoptosis and other basal functions at the microscopic level as well as being relevant to individual mammalian development at the macroscopic level (Bharadwaj et al., 2020). During physiologically conditioned osteogenesis, IL-6 family cytokines activate osteogenic differentiation and extracellular matrix synthesis of osteoblasts by stimulating Stat3 phosphorylation via binding to glycoprotein 130 (gp130), including IL-6, IL-11, oncostatin M (OSM), etc (Sims, 2016). Meanwhile, other non-receptor tyrosine kinases have been reported to activate Stat3, such as the Src kinase family, including Src, Lck, Hck, Lyn, and Fyn (Silva, 2004). Notably, in some specific circumstances, the intranuclear accumulation of unphosphorylated Stat3 (U-Stat3) can still regulate the cytoplasmic accumulation of cytokines that promote the expression of RANTES, which is an essential mediator of inflammation and do not respond directly to phosphorylated Stat3 (p-Stat3) (Yang et al., 2007). Since Stat3 can be activated directly or indirectly by a complex network of cellular signaling pathways and has a wide range of downstream effectors, the value of Stat3 in bone-related diseases requires further investigation.

## The Role of Signal Transducer and Activator of Transcription 3 Signaling Pathway in Bone-Related Diseases

### Signal Transducer and Activator of Transcription 3 Signaling Pathway in Osteoarthritis

OA is a chronically retrogressive disease typified by the release of inflammatory factors, cartilage erosion, osteophyte formation, and invasion of subchondral bone vessels, frequently accompanied by synovitis and pain (Hu et al., 2021a; Xue et al., 2021; Yajun et al., 2021). In the pathological microenvironment of OA, IL-6 induces a diminished synthesis of extracellular matrix proteoglycans in cartilage, while stimulating matrix metalloproteinases (MMPs) to disintegrate proteoglycans into the extracellular environment (Latourte et al., 2017). *In vivo* and *in vitro* experiments demonstrated that it is the activation of Stat3 in chondrocytes that induces cartilage destruction and osteophyte formation in OA, but not ERK1/2 (Latourte et al., 2017). By examining clinical cartilage specimens, Liang et al. revealed that retinoic acid receptor-related orphan receptor-*α* (ROR*α*) was positively correlated with the severity of OA and ROR*α* restored chondrocyte type II collagen (Col-2) and aggrecan expression by reversing the IL-6-induced increase in p-Stat3 levels (Liang et al., 2021).

Macrophage polarization in OA is under the regulation of multiple environmental irritants. The involvement of M1-type macrophages promotes chondrocyte damage and synovial inflammation in OA with subsequent release of tumor necrosis factor *α* (TNF*α*), IL-1*β* and IL-6 (Zhou et al., 2019). When Stat3 expression and phosphorylation are restrained by interferon-*γ* (IFN-*γ*), macrophages are stuck in M1 type and secrete massive levels of IL-1*β* and TNF*α* into the cell supernatant (Tian et al., 2021). Furthermore, extracellular vesicles (EVs) from mesenchymal stem cells (MSCs) promote chondrocytes proliferation, migration and anti-apoptosis by facilitating the switch from Stat3 to p-Stat3 under hypoxic conditions, which is closely analogous to the osteo-chondrogenic environment under physiological conditions (Rong et al., 2021).

### Signal Transducer and Activator of Transcription 3 Signaling Pathway in Osteoporosis

Over-activation of osteoclasts disrupts bone homeostasis and leads to osteoporosis, with primary fractures resulting from low bone mineral density (BMD) being the primary risk of disability and death in the elderly (Kirk et al., 2020; Mei et al., 2020). In the dialogue between osteoclasts and osteoblasts, receptor activator of nuclear factor κ B ligand (RANKL) is not only vital for osteoclast proliferation and formation, but also negatively modulates the osteogenic differentiation of MSC (Chen et al., 2018; Chen et al., 2020; Hu et al., 2021b). RANKL facilitates the expression of the downstream osteoclast marker NFATc1 and decreases tartrate-resistant acid phosphatase (TRAP) -positive cells by stimulating the Stat3 pathway (Li et al., 2021). Coincidentally, Yang et al. found that Stat3 can actuate NFATc1 transcription by binding to its promoter, which was similarly concluded in the experiments with the JAK2/Stat3 inhibitor AG490 *in vitro* and gene deletion of Stat3 in osteoblast(Yang et al., 2019). Thus, the RANKL-Stat3-NFATc1 axis may play a pivotal position in RANKL-induced osteoclast overactivation as a therapeutic target for osteoporosis.

On the other side of the disorder of osteohomeostasis, the engagement of Stat3 in osteoblast bone formation is likewise crucial. First, Stat3 upregulates Runx2 transcriptional activity, induces alkaline phosphatase (ALP) activation and calcium nodule mineralization by binding to the osteogenesis-associated transcription factor Runx2 (Xu et al., 2022). The subsequent ChIP experiments reveal that Stat3 directly binds to the promoter of the late osteogenic marker osteocalcin (OCN) and further enhances this effect upon the incorporation of Runx2 (Xu et al., 2020). Simultaneously, the synergistic effect of Stat3 and Runx2 on OCN promoter activity could be reversed by AG490 (Xu et al., 2020). The foregoing data suggest that Stat3 boosts osteoblast differentiation by enhancing OCN transcription through interaction with Runx2, ultimately reversing the bone loss caused by estrogen deficiency.

### Signal Transducer and Activator of Transcription 3 Signaling Pathway in Skeletal Development and Repair

Stat3 is generally recognized as an intracellular effector activated by inflammatory disorders in most diseases, but the latest evidences show that Stat3 is revealed to be an integral part of chondrogenesis and skeletal development (Yadav et al., 2021; Liu et al., 2022). The deletion of Stat3 in MSC and pre-osteoblasts leads to Autosomal dominant hyperimmunoglobulin E syndrome (AD-HIES)-like cranial deformities, significant reduction in cortical bone thickness and systemic osteoporosis, but not osteoclasts (Zhou et al., 2021). Transcriptome analysis revealed that several osteoblast-associated genes, including Dlx5, were down-regulated after Stat3 deletion (Zhou et al., 2021). Mechanistically, there are two Stat3 binding sites on Dlx5, and Stat3 drives Dlx5 transcription through direct binding and upregulation of promoter activity (Zhou et al., 2021). Similarly, Knockout Stat3 mice in osteocytes exhibit lower bone mass, decreased bone formation index, and diminished mechanical load-induced bone formation, indicating that Stat3 is a pivotal medium for osteocytes responding to mechanical stress (Corry et al., 2019).

**FIGURE 1:**
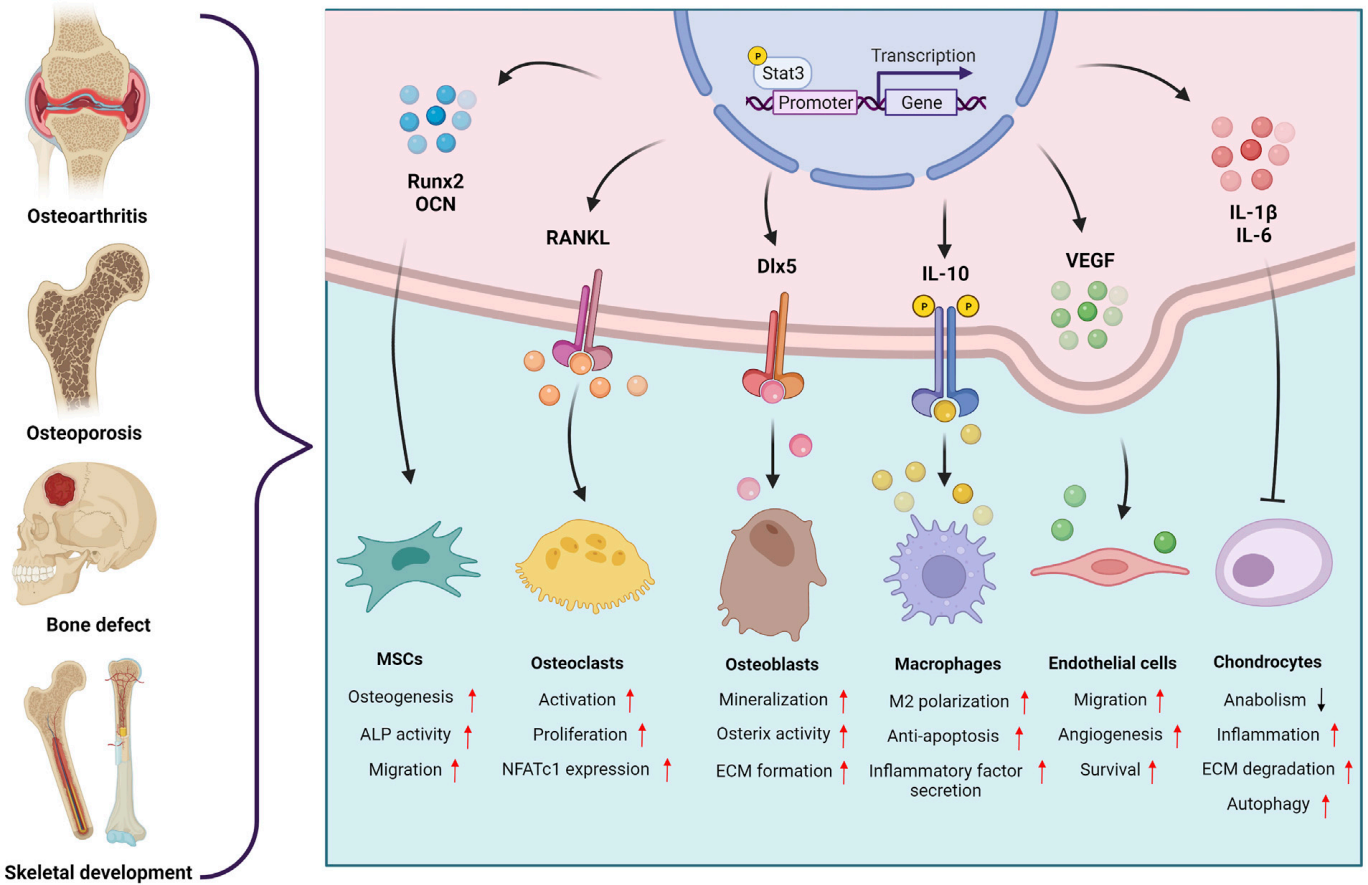
The regulatory role of the Stat3 signaling pathway in the microenvironment of bone-related diseases. Created with BioRender.com.

Bone repair and regeneration are regulated by cytokines expressed locally in the skeletal microenvironment as well as those elevated locally and systemically under inflammatory conditions (Damerau et al., 2020; Xiong et al., 2020; Lin et al., 2022). Multiple studies have shown that Stat3 influences bone repair progression by responding to a sophisticated regulatory network. In large-scale bone defects and delayed fracture healing, phosphorylation-activated Stat3 increased the rate of bone regeneration at the defect site by enhancing MSC osteogenic differentiation and vascularization (Yu et al., 2019; Chen et al., 2021). Coupling of angiogenesis and osteogenesis is imperative for post-traumatic bone regeneration, the activation of Stat3 in vascular endothelial cells would facilitate migration and angiogenesis, which could be a new way to exploit therapy for fracture and osteonecrosis (Chim et al., 2015; Gu et al., 2021). The aforementioned studies shed a light on the regulatory mechanisms and the great therapeutic potential of Stat3 (Figure 1). Therefore, hopes are pinned on targeting Stat3 for the cure of bone-related diseases.

## Target Signal Transducer and Activator of Transcription 3 Signaling Pathway for Bone-Related Diseases

### Synthetic Compounds

Current therapeutic regimens targeting Stat3 are mainly achieved through direct regulation phosphorylation, dimerization, DNA binding activity and nuclear translocation of Stat3 (Siveen et al., 2014). As shown in Table 1, scientists have investigated different types of compounds to modulate Stat3. Stattic is a selective inhibitor of Stat3 that effectively inhibits its activation and nuclear translocation (Schust et al., 2006). In chondrocytes and explants, Stattic revered IL-6-induced over-expression of MMPs and demonstrated superior efficacy than the anti-IL-6-receptor neutralizing antibody in DMM mice (Latourte et al., 2017). Analogously, Li et al. revealed that Stattic inhibited RANKL-mediated osteoclastogenesis and ovariectomy-induced bone loss in a dose-dependent manner, implying that Stattic represents a new class of osteoclast inhibitors (Li et al., 2018). In the iodoacetic acid-induced OA model, Stat3 dimerization and DNA binding were blocked by STA-21, which alleviated joint pain and inflammatory damage in rats (Lee et al., 2018). Nitazoxanide (NTZ) has broad-spectrum antibacterial and antiprotozoal capabilities, yet researchers have found that Stat3 binding to the NFATc1 promoter can be attenuated by NTZ to rescue bone loss, which opens up new scenarios for NTZ applications (Li et al., 2021). The same conclusion was also confirmed in the study on BCI hydrochloride (BCI) (Cai et al., 2021).

**TABLE 1:** Different types of targeting Stat3 signaling pathway and their biological functions.

Classification	Name	t	Biological Functions	References
Synthetic compounds	Stattic	OA, OP	Inhibit osteoclast activation and IL-6-induced chondrocyte apoptosis	Latourte et al. (2017), Li et al. (2018)
	STA21	OA	Detect MIA-induced joint pain and cartilage damage	Lee et al. (2018)
	Nitazoxanide	OP	Suppress Stat3 phosphorylation and reduce Ca2+ fluorescence intensity	Li et al. (2021)
	BCI	OP	Diminish NF-κB signaling and RANKL-induced osteoclast differentiation	Cai et al. (2021)
	Tofacitinib	OA	Minimize chondrogenic hypertrophy and inflammatory factors	Chiu et al. (2021)
	AG490	Skeletal development, Bone defect	Reduce MSC osteogenic matrix mineralization and triggers bone loss	Yu et al. (2018), Zhou et al. (2021)
	Colivelin	Skeletal development	Facilitate Stat3 phosphorylation and bone formation in tail-suspended mice	Zhou et al. (2021)
Natural compounds	Alantolactone	OA	Selective restraint of Stat3 nuclear translocation and regulate chondrocyte autophagy	Pei et al. (2021)
	Lycopene	OA	Restrain the expression of COX-2 and iNOS and restore ECM reconstruction in chondrocytes	Zhan et al. (2021)
	Angelicin	OA	Upregulate CD9 expression to polarize macrophages toward M2 type and mitigate OA development	Tian et al. (2021)
	BDMC	OP	Enhance the expression of calcium deposition and osteogenic markers in MSC at the transcriptional and translational levels	Wei et al. (2022)
	Icariin	OP	Promote alveolar bone formation in OVX rats by binding of Stat3 to the OCN promoter	Xu et al. (2020)
	Catalpol	Bone defect	Activate the JAK-Stat3 axis to drive BMSC-mediated angiogenesis *in vivo* and *in vitro*	Chen et al. (2021)
	Poria cocos polysaccharide	Fractures	Inhibits phosphorylation activation of MAPK and Stat3 signaling pathways to reduce osteoclast activity	Song et al. (2018)
RNA and proteins	miR-216a-5p	OA	Accelerate chondrocyte proliferation, migration and anti-apoptosis by directly targeting the 3′-UTR of JAK2	Rong et al. (2021)
	miR-151a-3p	OP	Lower the BMD and biomechanical parameters of the femur to boost the OP process	Fu et al. (2020)
	Leptin	OA	Modulate TLR4 expression by activating CD14 through the JAK2/ Stat3 signaling pathway	Jiang et al. (2021)
	MYDGF	OP, Bone defect	Elevate Stat3 phosphorylation on S727 and calcium mineralization in cranial osteoblasts	Xu et al. (2022)

In addition, Stat3 signaling pathway can be indirectly suppressed by blocking upstream regulators such as IL-6, JAK and EGF (Yu et al., 2018; Di Benedetto et al., 2021; Zhou et al., 2021). AG490 and tofacitinib are JAK tyrosine kinase inhibitors, which are widely studied as JAK2 inhibitors in immune and inflammatory diseases (Chen et al., 2019). Notably, tofacitinib, manufactured by Pfizer, has been approved by the FDA for the treatment of rheumatoid arthritis, ankylosing spondylitis, psoriasis, and ulcerative colitis (Pfizer, 2021). Articular cavity injection of tofacitinib promotes miR-149-5p expression to restore cartilage homeostasis and downregulates the JAK/Stat3/IL-6/TNF-*α* axis to arrest cartilage hypertrophy in human chondrocyte lines (Chiu et al., 2021). The inhibition of Stat3 phosphorylation in osteoblasts by AG490 will result in poor prognosis of bone defects and dysosteogenesis, the administration of the Stat3 activator colivelin partially rescues this alteration restoring ALP and Runx2 expression (Yu et al., 2018; Zhou et al., 2021). Overall, these findings suggest that bone-related diseases could be healed by directly or indirectly targeting Stat3, but further clinical research is still needed.

### Natural Compounds

Various natural compounds appear to be efficacious in bone-related diseases and hold promise as a succedaneum for synthetic compounds as the next generation of therapeutic agents. Alantolactone and lycopene inhibit IL-1*β*-induced activation of NF-κB and Stat3 and attenuate chondrocyte autophagy and extracellular matrix (ECM) degradation (Pei et al., 2021; Zhan et al., 2021). Alantolactone at 2 mg/kg alleviated medial meniscus wear and MMP13-positive cell counts *in vivo* (Pei et al., 2021). By focusing on macrophages in post-trauma OA, Tian et al. found that angelicin could adjust the M1/M2 ratio in synovial tissue and protect articular cartilage through the CD9/gp130/Stat3 pathway (Tian et al., 2021). Angelicin upregulates the expression and phosphorylation of Stat3, which further significantly promotes the expression of Arg-1 and CD206 (Tian et al., 2021). In addition, Poria cocos polysaccharide also inhibited Stat3 signaling pathway in osteoclasts to attenuate RANKL-induced osteoclastogenesis, which may open new doors for the therapy of pathological fractures (Song et al., 2018).

Unlike chondrocytes or osteoblasts, Stat3 activation plays a positive role in OP and bone repair. Bisdemethoxycurcumin (BDMC) and Icariin increase the level of Stat3 phosphorylation in MSC, while facilitating the expression of osteogenic differentiation markers at the transcriptional and translational levels, such as ALP, Runx2, OCN, osteopontin (OPN), collagen 1-*α*1 (Col1-*α*1), etc. (Xu et al., 2020; Wei et al., 2022). In addition, catalpol promotes phosphorylation of Stat3 and nuclear translocation of p-Stat3, which enhances bone healing capacity and vascular endothelial growth factor (VEGF) secretion (Chen et al., 2021). Micro-CT and angiography demonstrated significant increases in the area of bone regeneration and the number of blood vessels at the site of cranial defects in rats after intraperitoneal injection of catalpol (Chen et al., 2021). However, future studies are needed to provide further evidence for the application of natural compounds in bone-related diseases.

### RNA and Proteins

RNA and proteins are critical regulators in mammals, with multiple reports suggesting that they mediate bone-related diseases by targeting Stat3. EVs-derived miR-216a-5p promotes chondrocyte proliferation, migration and anti-apoptosis by restraining JAK2 expression and Stat3 phosphorylation (Rong et al., 2021). Luciferase reporter gene assays reveal that miR-216a-5p directly targets the 3′-UTR of JAK2, which leads to the blockage of Stat3 phosphorylation (Rong et al., 2021). Interestingly, miR-151a-3p was overexpressed in the femur of OP and negative regulation of MC3T3-E1 osteogenic differentiation by the JAK2/Stat3 signaling pathway was identified (Fu et al., 2020).

Leptin is the most secreted adipokine in white adipose tissue and it also seems to trigger obesity-related OA via Stat3 (Cordero-Barreal et al., 2021). Elevated leptin in serum induces activation of Stat3 in cartilage and leads to pathological activation of CD14/TLR4, which further provokes obesity-associated inflammation and MMP-13 expression (Jiang et al., 2021). Analogously, the myeloid-derived growth factor (MYDGF) increases Stat3 phosphorylation on S727 (Xu et al., 2022). MYDGF promotes bone defect healing by promoting ALP activity and mineralization in primary cranial osteoblasts, in which Stat3 activation plays a pivotal role (Xu et al., 2022). Nevertheless, the road from targeting Stat3 to mature therapies for bone-related diseases remains long, and therapeutic applications for either activating or inhibiting Stat3 have limitations.

## Conclusion and Future Perspectives

Stat3 is a transcriptional regulator and is activated by a broad range of cytokines in the bone microenvironment. Increasing reports indicate that Stat3 exerts regulatory effects via multiple pathways affecting bone-related diseases, including inflammatory stimulation, cartilage degradation, osteoclast activation, osteoblast differentiation, and macrophage polarization. Activation of Stat3 in MSC or vascular endothelial cells could promote their proliferation and differentiation in favor of bone defect repair, while on the other hand inhibition of Stat3 in chondrocytes and osteoblasts would alleviate OA and OP. However, the role of Stat3 in the regulation of bone-related diseases has not been clearly described, patients with bone-related diseases continue to cause great disturbance in their daily life. Current research supports the use of Stat3 as an emerging target for the management of bone-related diseases with exciting results.

Nonetheless, targeting Stat3 to achieve the treatment of orthopedic diseases still has a long way to go from bench to bedside. Targeting Stat3 as a therapeutic agent is like a double-edged sword, with unpredictable potential toxicity and side effects while being highly effective. As a very dense tissue in the human body, the effective and targeted delivery of bone still faces great challenges, and how to achieve precise drug delivery will be the direction of future research. Picking the suitable period of treatment is also extremely valuable, as the choice of timing of drug interventions often correlates with preferable efficacy. After overcoming these challenges, targeting Stat3 has the potential to be a promising therapeutic option for bone-related diseases.
